# Strength Training and Posture Correction of the Neck and Shoulder for Patients with Chronic Primary Headache: A Prospective Single-Arm Pilot Study

**DOI:** 10.3390/jcm14155359

**Published:** 2025-07-29

**Authors:** Jordi Padrós-Augé, Henrik Winther Schytz, Karen Søgaard, Rafel Donat-Roca, Gemma Victoria Espí-López, Bjarne Kjeldgaard Madsen

**Affiliations:** 1 Department of Physiotherapy, University of Vic—Central University of Catalonia, Campus UManresa, 08242 Manresa, Spain; 2 Sport, Exercise and Human Movement, University of Vic—Central University of Catalonia, 08500 Vic, Spain; 3 Danish Headache Centre, Department of Neurology, Faculty of Health Sciences, Glostrup Hospital, 2600 Copenhagen, Denmarkbjarne.kjeldgaard.madsen@regionh.dk (B.K.M.); 4 Department of Sports Science and Clinical Biomechanics, University of Southern Denmark, 5230 Odense, Denmark; 5 Department of Clinical Research, University of Southern Denmark, 5230 Odense, Denmark; 6 Department of Physiotherapy, Faculty of Physiotherapy, University of Valencia, Gasco Oliag St, 5, 46010 Valencia, Spain; 7 Exercise Intervention for Health Research Group (EXINH-RG), Faculty of Physiotherapy, University of Valencia, Gasco Oliag St, 5, 46010 Valencia, Spain

**Keywords:** migraine, tension-type headache, chronic headache, exercise, strength training, non-pharmacological treatment

## Abstract

**Background:** Few studies have examined exercise-based treatments for migraine and tension-type headache (TTH), and even fewer have focused on strength training and chronic headache, as these present greater challenges. **Objectives**: This study aimed to evaluate the effectiveness of a group-based neck and shoulder strength training intervention combined with postural correction for patients with chronic headache. **Methods**: This prospective, single-arm, uncontrolled pilot study with a pre–post design included patients with chronic migraine (*n* = 10) and TTH (*n* = 12) who participated in an 8-week group-based program consisting of neck and shoulder strength training three times per week, along with instructions for postural correction. The primary outcome was change in headache frequency. Secondary outcomes included changes in the intensity and duration of headache, number of days of analgesic use, and functionality. **Results**: In total, 22 patients completed the intervention and were included in the analysis. Headache frequency decreased at follow-up for the overall group (r = 0.531; *p* = 0.014). In-depth analysis showed that 45% of participants experienced an average reduction of 38% in headache frequency. Additionally, large to moderate effect sizes were observed for the secondary outcomes. **Conclusions**: This is the first study to introduce a group-based exercise program targeting the neck and shoulder muscles, combined with postural correction and standard pharmacological treatment, for patients with chronic primary headache. It was found to be a safe, well-tolerated, useful, and promising intervention for improving headache frequency, duration, and functionality.

## 1. Introduction

Danish national guidelines highlight a significant need for further documentation of non-pharmacological interventions for the treatment of migraine and tension-type headache (TTH) [1]. Exercise-based treatments may affect the central and peripheral mechanisms involved in migraine and TTH. Although the two types of headache share some similarities, their pathophysiology also exhibits key differences that remain complex and not fully understood [2]. One shared feature is the presence of neck pain, which is a prognostic factor for the progression of headaches [3,4]. In TTH, increased pericranial sensitivity appears to play a role in activating the cervical trigeminal system whereas, in migraine, it typically follows episodes [5]. Muscle tenderness is more pronounced in chronic headaches than episodic ones, and patients with chronic neck pain exhibit levels of tenderness similar to those with chronic headache. This suggests that increased muscle tenderness is both a trigger and a manifestation, particularly in chronic migraine [6]. Additionally, impaired neck function can lead to neck pain, which is a poor prognostic factor for migraine [7].

Previous studies have reported differences in the architecture and function of the cervical and shoulder muscles in patients with migraine and TTH, indicating greater weakness and instability in this region from a functional perspective [8,9]. A link between muscle tenderness and muscle function (e.g., decreased force steadiness and an altered neck extension/flexion ratio) has been documented in patients with chronic and frequent episodic TTH [8]. Low-load cervical training has been explored in both chronic TTH and migraine, typically in combination with other treatments such as relaxation or manual therapy [10,11]. Shoulder strength training has been studied in patients with chronic and frequent episodic TTH, but it has shown limited impact on headache outcomes, with no measurable strength gains, suggesting suboptimal training intensity [12,13]. These findings highlight the need for more tailored, supervised interventions, as well as increased attention to confounding factors such as adherence and compliance.

While strength training aims to increase neck strength and normalize muscle function, posture correction seeks to reduce the relative muscular load. For example, forward head posture can contribute to continuous loading of the neck muscles, and correcting it may reduce that load [14]. Therefore, examining how a combination of low-load neck exercises, shoulder strength training, and postural corrections can influence chronic primary headaches is warranted.

Based on trigeminal convergence theory, this intervention aims to reduce afferent nociception input by lowering muscle load through postural correction and improving neck muscle function through motor control and strength training. Shoulder strength training at 70–80% of one-repetition maximum (1RM) has been associated with changes in muscle function [15], which typically occur in the medium- to long-term and are dependent on training intensity. In contrast, low-load neck muscle training improves balance and function in the upper cervical segments [16]. Thus, combining these modalities may enhance the effects of pharmacological treatment, reducing trigeminospinal central sensitization and headache [17].

Combining pharmacological and non-pharmacological strategies may enhance outcomes for migraine patients, although this approach has not yet been studied. These effects may extend beyond headache symptoms to functional impairments in patients’ ability to perform daily life activities, which are often reported by people with chronic headaches [18].

Monoclonal antibody (CGRP) treatments target cranial blood vessel vasodilatation and neuroinflammation. Strength training may complement these therapies by reducing peripheral nociception from the neck muscles [19]. Onabotulinum toxin A (BoNT/A) acts on both peripheral tissues and central mechanisms [20], and strength training may enhance its effects. Moreover, strength training might help to prevent common side effects, such as neck pain or neck weakness [21].

The present study investigates the effects of exercise as a standalone physical therapy intervention using a previously unexplored combination of active interventions. The primary aim of this study is to assess the effects of supervised group strength training and posture correction on migraine and TTH. Given the lack of prior studies on exercise interventions in patients with refractory chronic primary headaches, we opted to conduct a pilot study focused on adherence and tolerability as key outcomes. We hypothesized that this intervention would be well tolerated and lead to reductions in headache.

## 2. Materials and Methods

### 2.1. Design

A prospective, single-arm, uncontrolled pilot study with a pre–post design was performed at the Danish Headache Center (DHC) from October 2023 to January 2024 as part of clinical operations (clinicaltrials.gov ID: NCT06112587; registration date: 10 October 2023). Data was collected at the DHC, where exercise sessions and assessments were conducted by a physiotherapist (JPA).

### 2.2. Participants

Patients diagnosed with chronic migraine or TTH by a neurologist at the Danish Headache Center (DHC), according to the third edition of the *International Classification of Headaches Disorders* (ICHD-3), were invited to participate in a group exercise program during the recruitment period. Ethical approval was obtained (F-23036562), and all participants provided written informed consent in accordance with the Declaration of Helsinki.

### 2.3. Inclusion and Exclusion Criteria

Participants were included according to the following criteria: (i) aged 18–65 years; (ii) diagnosis of chronic migraine, frequent episodic TTH, or chronic TTH; (iii) stable CGRP or BoNT-A medication (at least two doses of the same pharmacological treatment) for migraineurs; and (iv) at least 5 days of headache per 14-day period at baseline. Exclusion criteria included pregnancy; post-traumatic headache or headache likely associated with trauma; significant psychiatric comorbidities (e.g., severe depression); medication overuse headache; severe cervical/shoulder osteoarthrosis or disc herniation; and other neurological conditions (e.g., multiple sclerosis). All patients who attended the DHC during the four weeks prior to the baseline data collection period were invited to participate.

### 2.4. Outcomes and Data Collection

**Figure 1:**
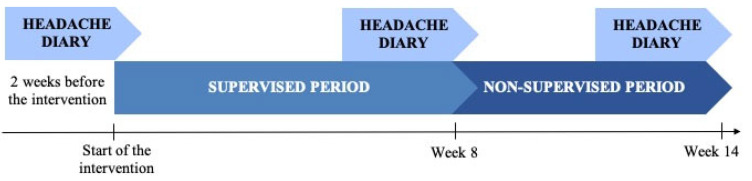
Study diagram.

The primary outcome was headache frequency (days with a headache per 14-day period). Patients completed a two-week headache diary at baseline (T0), at the end of the supervised period (weeks 7–8; T1), and after a non-supervised period (weeks 13–14; T2) (Figure 1). Secondary outcomes from the diaries included headache intensity, measured using an 11-point numerical rating pain scale (NRS); headache duration, measured as hours with a headache per 14 days; analgesic use, measured as days with analgesic use per 14 days; and neck pain frequency and intensity. In addition, the Patient-Specific Functional Scale (PSFS) was administered to assess functionality [22]. Finally, adherence and self-reported exercise intensity were assessed using an exercise diary.

### 2.5. Headache Diary and Questionnaires

Patients were asked to complete baseline, endpoint, and two-week follow-up headache diaries. Recorded data included headache frequency, maximum and average intensity, duration, medication intake, and neck pain [23,24]. Intensity was registered independently from headache and neck pain on an 11-point NRS (0 = no pain, 10 = worst pain). Medication intake was recorded as the number of days with analgesic use. In addition, the area under the curve (AUC)—a more global indicator of hours with headache and intensity—was calculated as ∑ (intensity ∗ duration) for each 2-week period.

Exercise diaries included data on acute headache and neck pain before and after each exercise session. The physiotherapist reviewed the initial sessions to ensure accurate reporting without influencing patient entries. Functionality was assessed using the PSFS, a valid, reliable, and responsive self-reported outcome measure for patients with musculoskeletal, neurological, and chronic pain conditions [25]. The PSFS asks patients to identify up to five daily activities impaired by headache and rate the associated functional limitations on an 11-point scale. A final score is calculated as the average of these ratings.

### 2.6. Adherence, Self-Perceived Intensity, and Compliance

Adherence and compliance were documented during the 8-week program and the follow-up period. Adherence was calculated as the number of sessions performed divided by the number of sessions planned. Compliance was assessed as the proportion of sessions performed at a perceived exertion rate (RPE) of 7–9 on a 10-point scale (1 = no effort, 10 = maximum effort). After each session, patients recorded their RPE. Compliance was calculated as follows:

$$\mathrm{Compliance}=\frac{\mathrm{Number}\;\mathrm{of}\;\mathrm{sessions}\;\mathrm{with}\;\mathrm{RPE}\;\mathrm{range}\;\mathrm{achieved}}{\mathrm{Number}\;\mathrm{of}\;\mathrm{sessions}\;\mathrm{performed}}$$

### 2.7. Intervention

The 8-week intervention included one weekly supervised group session and two home sessions, supported by posture and ergonomic guidance [10,12,26]. Groups of 4–6 patients (mixed migraine and TTH) trained for 45–60 min per session. In-person sessions were conducted in an environment with controlled lighting and minimal noise to reduce sensory triggers and ensure participants’ comfort. Exercises were based on validated protocols for TTH, migraine, and neck pain [12,16]. Patients used TheraBands^®^ (Akron, OH, USA) with tailored resistance, and the physiotherapist adjusted the participants’ exercise technique and intensity to meet their individual needs during the supervised period. Patients received a support guide with guidelines for postural correction and ergonomics, including instructions for performing exercises and progressing exercise intensity, as well as an exercise diary. Participants were asked to record the date, time, and place of each exercise session, headache pain before exercise and 2 h after, postural corrections performed, and the RPE score for each session (Supplementary Materials). At the end of the intervention, patients were encouraged to continue training until the follow-up assessment.

### 2.8. Data Analysis

Descriptive statistics are reported as absolute frequencies and percentages for categorical variables and medians and ranges for continuous variables. Data distribution was assessed using the Shapiro–Wilk test. Group-level comparisons for continuous variables were performed using the Mann–Whitney U test due to non-normal data distribution and the small sample size (*n* < 30). For within-group comparisons, the Wilcoxon signed-rank test was applied. Fisher’s exact test was used for categorical variables. Results are presented as changes from baseline (T0) to the end of the supervised period (T1) and from baseline (T0) to the end of the program (T2). A descriptive analysis of adherence and compliance was also performed. A significance level of *p* < 0.05 was established for all statistical tests. Finally, effect sizes (r) were calculated for within-group comparisons using the formula r = Z/$\sqrt{N}$, where Z is the test statistic from the Wilcoxon signed-rank test and N is the sample size. Effect sizes were interpreted as small (r = 0.1–0.3), moderate (r = 0.3–0.5), or large (r > 0.5). The analysis was performed using JASP 0.19.1 (JASP Team, 2024).

This study was reported in accordance with the CONSORT extension for pilot and feasibility trials to ensure transparency and methodological clarity [27].

## 3. Results

**Figure 2:**
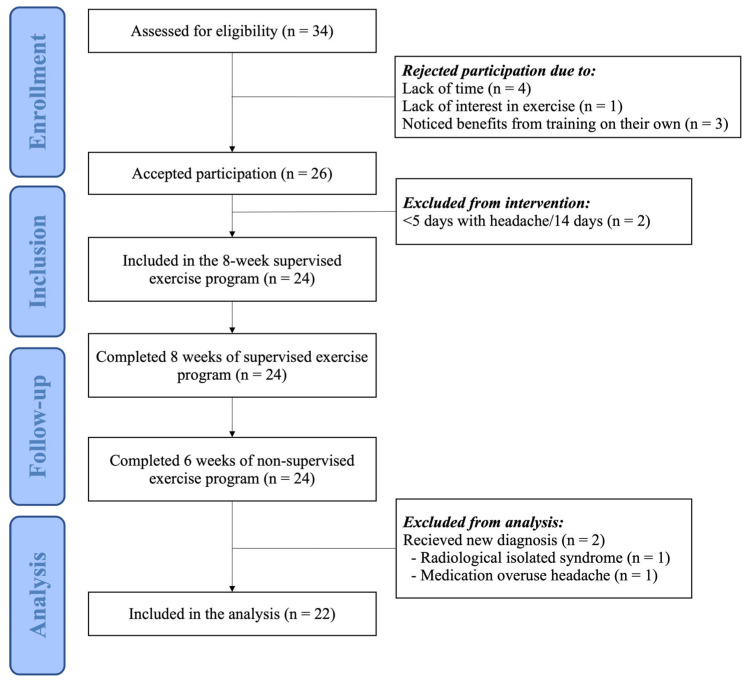
Flowchart of the participants in the study.

A total of 24 patients completed the 2-week headache diary and participated in the exercise program. All patients completed the 14-week exercise program, the 8-week supervised period, and the 6-week non-supervised period. Of these patients, 11 had migraines and 13 had TTH (Figure 2). All headache diaries and questionnaires were collected. One participant was excluded from the analysis due to medication overuse headache (*n* = 1). Another participant received a new diagnosis of radiologically isolated syndrome during the intervention period. As the potential effect of this diagnosis on the participant’s headache was unclear, they were excluded from the analysis (*n* = 1). However, this patient did experience improvement during the intervention, and their data can be shared upon reasonable request. The baseline characteristics of the participants are presented in Table 1. Intervention details, including adherence, compliance, and reasons for missing sessions, are summarized in Table 2. Data on headache, medication, neck pain, and functionality at T0, T1, and T2 are presented in Table 3.

### 3.1. Primary Outcome

The primary outcome was headache frequency. A statistically significant reduction was observed at follow-up compared to baseline for the overall group (*p*-value = 0.014) (Table 4). In-depth analysis showed that 12 patients did not experience an improvement in headache frequency, while 10 patients showed a significant reduction according to International Headache Society standards [28]. One patient experienced a ≥ 75% reduction, two patients experienced a ≥50% reduction, and seven patients experienced a > 30% reduction (median: −4 days/14-day period), representing a combined 38% reduction in headache for these patients. Results stratified by headache type are presented in Table 4.

### 3.2. Secondary Outcomes

A large effect size was observed for headache duration reduction from T0 to T2 (r = 0.562; *p* = 0.009). A 55% mean reduction in headache duration was shown in 12 patients, while no changes were found for the others. Similarly, the area under the curve (AUC) decreased by 22% from T0 to T2 for the overall group, with a significant change and a large effect size (r = 0.508; *p* = 0.018). In contrast, overall function improved significantly from T0 to T2 (*p* < 0.001), with a large effect size (r = 0.716). Finally, a moderate effect was found for neck pain frequency reduction from T0 to T2 (r = 0.425; *p* = 0.049).

### 3.3. Adherence and Compliance

Adherence and compliance are reported and summarized in Table 4. Average adherence was 73%, including 83% during the supervised period and 61% during the follow-up period. The main reason for missing sessions during the supervised period was sickness (60%), followed by severe headache (20%). During the non-supervised period, sessions were most often missed due to holidays (45%), followed by lack of time (15%) and sickness (15%). The first two weeks of the non-supervised period coincided with the Christmas holidays, and then adherence increased progressively until the final week of the follow-up period. Compliance (7–9/10 RPE) during the supervised period exceeded 70% for all weeks except for the first (47%), during which patients were familiarizing themselves with the exercise plan. During the non-supervised period, patients who exercised achieved an average compliance of up to 94%, and none of them exceeded the target intensity (10/10 RPE = 0%).

## 4. Discussion

This is the first study to examine a group-based exercise intervention in chronic migraine patients on stable medication (BoNT-A or CGRP), combining specific exercises not previously implemented together. The results indicate a median reduction of 4 headache days per 14-day period, although not all participants experienced improvement. While 12 patients had a 55% reduction in headache duration, others showed no change. The AUC decreased primarily due to reductions in frequency and duration, with no significant change in intensity. These findings are promising for a subset of patients and highlight the need to identify factors associated with treatment response.

Functional improvements were observed, with 67% of self-reported activities on the PSFS showing clinically meaningful gains. Strength training may enhance patients’ ability to better tolerate relative workloads and increase their capacity to perform daily activities without triggering a headache.

The compliance analysis suggests that eight weeks of one session supervised by a physiotherapist and two home sessions per week are sufficient to teach the exercise program. Adherence results suggest that additional support, such as reminders or extended supervision, may benefit some patients. Considering the locus of control at the end of the supervision period may help in identifying which patients require extended supervision, monitoring, or reminders to maintain adherence during the follow-up period [29]. Adding other interventions, such as cognitive behavioral therapy or motivational interviewing, may increase functional status by assessing fear avoidance and sedentary behaviors, which were not assessed in this study but could potentially influence adherence and functionality [30,31].

This intervention is notably time-efficient, requiring only 15–20 min per session for the strength training exercises, which is much less than the 30–45 min typically required for aerobic training. This could be particularly beneficial for chronic migraine patients who struggle with longer sessions. Strength training may offer similar or even superior benefits for this population, with fewer weekly sessions over longer periods [32].

Given the chronic nature of the conditions experienced by this sample and the time constraints they face, this approach appears highly feasible. Group training in a controlled, comfortable environment may further support adherence, and group settings may improve psychological well-being by fostering social support.

The mechanisms through which shoulder strength training can influence TTH and migraine should be further investigated in a randomized controlled trial. Furthermore, exploring how the intervention affects muscle function could clarify the role of muscles in TTH and migraine. Poor neck and shoulder muscle function may contribute to fatigue in these muscle groups during daily activities.

Improved muscle function may reduce fatigue, enhance daily activity tolerance, and lower nociceptive input to the trigeminal system, thereby decreasing headaches [33]. In connection with this study, a separate manuscript will present findings on muscle function and muscle tenderness.

The results of this study should be interpreted with caution due to several limitations. First, the absence of a control group represents the main limitation in assessing the effectiveness of the intervention. Although medication effects were partially controlled through stable use and a conservative timeline, pharmacological influence cannot be excluded. Second, the use of a 14-day headache diary may have been limiting, especially for patients with episodic headaches, due to variability. However, this is less problematic in patients with chronic, pharmacologically resistant headaches. Third, the intervention’s duration may have been too short to capture full effects, particularly for patients who may need more time to notice changes. Previous studies using the same exercises have conducted evaluations over approximately 20 weeks [34]. Exercise is a time-efficient strategy that yields greater benefits when sustained and progressively intensified over time. To maximize its effectiveness, a longer intervention period with long-term follow-up is recommended.

For these reasons, future research should include a larger sample size, a control group, and longer follow-up. Maintaining appropriate monitoring and assessing participants’ perceptions of the exercises’ usefulness are also essential for evaluating their implementation. Additionally, incorporating a questionnaire to assess medication tolerance in combination with the intervention could provide valuable insights. Notably, some patients reported improved tolerance and fewer side effects from medication during the intervention period.

## 5. Conclusions

Group-based strength training of the shoulder and neck muscles, combined with postural correction, is a safe and well-tolerated non-pharmacological intervention for patients with chronic primary headache. Although patients with chronic headaches were able to comply with the intervention, headache days were the greatest intrinsic barrier.

This intervention increases functionality and may reduce the frequency and duration of headaches in patients with chronic migraine and chronic tension-type headaches.

Patients with chronic primary headaches were able to follow the strength training program after being partially supervised for 8 weeks. The results are promising for both patients with chronic migraine and those with chronic and frequent episodic TTH, demonstrating a reduction in days with a headache per month and improvements in functional status. However, further studies with robust methodologies should be performed.

## Figures and Tables

**Table 1:** Baseline characteristics of the participants.

		Sample	TTH	Migraine	*p* (*)
Variables		*n* (%)	*n* (%)	*n* (%)	
Sex *^a^*	Females	18 (81.8)	8 (66.7)	10 (100)	0.096
	Males	4 (18.2)	4 (33.3)	0 (0)	
		Mean ± SD	Mean ± SD	Mean ± SD	
Age		33.0 ± 9.6	29.9 ±8.1	36.6 ± 11.2	0.056
Years lived with headache		13.1 ± 10.5	8.4 ± 5.5	18.6 ± 13.2	0.037 *
Neck pain characteristics					
Presence of NP (n; %) *^ a^*		19; 86.4%	10; 83.3%	9; 90.0%	>0.999
Mean intensity of NP (0–10)		4.0 ± 1.3	4.4 ± 1.2	3.6 ± 1.3	0.174
Maximum NP intensity (0–10)		5.9 ± 2.0	6.3 ± 1.3	5.4 ± 2.4	0.218
Frequency (days with NP/14 days)		10.9 ± 3.3	10.6 ± 3.7	11.2 ± 3.2	0.754
Headache characteristics					
Average intensity (0–10)		4.9 ± 1.1	5.0 ± 0.9	4.7 ± 1.4	0.429
Maximum intensity (0–10)		7.3 ± 1.4	7.1 ± 1.2	7.5 ± 1.7	0.532
Frequency (days with headache/14 days)		11.7 ± 3.4	11.3 ± 3.8	12.1 ± 3.1	0.942
Duration (hours with headache in 14 days)		215.3 ± 123.8	220.7 ± 130.2	208.8 ± 122.3	0.661
Medication intake (days with meds/14 days)		4.23 ± 4.0	3.8 ± 5.0	4.7 ± 2.5	0.126
Pharmacological treatment for migraine Botox (n; months since first dose)*^ b^* Anti-CGRP (n; months since first dose)*^ b^*				4; 7.8 (5–11) 6; 6.7 (3–10)	
Overall function					
Patient-Specific Functional Scale		3.5 ± 1.5	3.6 ± 1.4	3.3 ± 1.7	0.488

TTH: tension-type headache; SD: standard deviation; NP: neck pain; anti-CGRP: monoclonal antibodies against calcitonin gene related peptide. *^a^* Fisher’s exact test was used for categorical variable comparisons. *^b^* Expressed by absolute values and the mean (minimum–maximum). * Between-group (migraine vs. TTH) statistically significant differences (significance level *p* < 0.05).

**Table 2:** Description of adherence and compliance.

	Supervised Period								Non-Supervised Period						Supervised Weeks 1–8	Non- Supervised Weeks 9–14	All
Weeks	1	2	3	4	5	6	7	8	9 *	10 *	11	12	13	14			
Adherence (%)																	
Sessions performed	91	91	92	77	79	89	79	65	44	41	62	67	73	77	83	61	73
Compliance (%)																	
RPE 7–9	47	73	82	84	83	78	86	90	100	95	100	91	87	91	78	94	85
RPE < 7	52	22	18	16	15	19	14	10	0	5	0	9	13	9	21	6	14
RPE = 10	2	5	0	0	2	3	0	0	0	0	0	0	0	0	1	0	1
Missed sessions **																	
N° of missed sessions	6	6	5	15	14	7	14	23	37	39	25	22	18	15	90	156	246
Reasons (*n*)																	
Severe headache	1	4	2	2	3	2	1	3	4	3	3	4	4	3	18	21	39
Work	3				1	3	2	3				1		1	12	2	14
Sickness	2	1	3	11	8	4	10	15	8	6	3	3	2	1	54	23	77
Holidays				2	1			2	22	25	15	9			5	71	76
Lack of time					1		1		1	2	2	2	9	8	2	24	26
Other		1							2	3	2	3	3	2	1	15	16

RPE: rate of perceived exertion; an RPE between 7 and 9 was considered compliant. * Weeks 9–10 coincided with the Christmas holiday period. ** The number of missed sessions accounts for all groups.

**Table 3:** Medians and ranges of headache-related outcomes, neck-pain-related outcomes, and functionality at baseline, weeks 7–8, and weeks 13–14 for the overall group and for each headache type.

	Baseline	Weeks 7–8	Weeks 13–14
Overall group (*n* = 22)			
Intensity	4.9 (2.8, 7.6)	5.1 (2.6, 7.3)	5.0 (0, 6.7)
Frequency	14 (5, 14)	13.5 (3, 14)	10.5 (0, 14)
Duration	262.5 (32, 336)	179 (37, 336)	134.5 (0, 336)
AUC	1208.9 (128, 2546.9)	923.6 (123.2, 2305)	623.5 (0, 2254.6)
Medication	4 (0, 8)	4 (0, 7)	3 (0, 7)
PSFS	3.2 (1.0, 6.2)	4.9 (1.8, 7.8)	5.5 (2.3, 8.8)
NP intensity	3.9 (2.3, 6.1)	3.6 (0, 7.4)	3.4 (0, 6.8)
NP frequency	10.5 (0, 14)	10 (0, 14)	8 (0, 14)
Migraine (*n* = 10)			
Intensity	4.4 (2.8, 7.6)	5 (2.6, 7.3)	5.3 (2.6, 6.4)
Frequency	13.5 (8, 14)	11.5 (8, 14)	9.5 (6, 14)
Duration	246.5 (32, 336)	204 (60, 336)	134.5 (20, 336)
AUC	1258.5 (128, 2546.9)	1134.8 (255, 2305)	612.8 (93.4, 1945.4)
Medication	4.5 (0, 8)	4 (0, 7)	3 (0, 7)
PSFS	2.7 (1, 6)	4.8 (1.8, 6.6)	5.0 (2.3, 7.6)
NP intensity	2.9 (2.4, 6.1)	2.7 (0, 5.6)	3.1 (0, 5.2)
NP frequency	12 (5, 14)	10.5 (0, 14)	8 (0, 14)
Tension-type headache (*n* = 10)			
Intensity	5.1 (3.2, 6.7)	5.2 (2.6, 7.3)	4.6 (0, 6.7)
Frequency	14 (5, 14)	14 (3, 14)	12.5 (0, 14)
Duration	286.5 (55, 336)	174.5 (37, 336)	140.5 (0, 336)
AUC	1136.6 (360, 2254.6)	837.9 (123.2, 2281.4)	579.0 (0, 2254.6)
Medication	2 (0, 7)	1.5 (0, 6)	1.5 (0, 6)
PSFS	3.4 (1.5, 6.3)	5.0 (3, 7.8)	5.9 (2.3, 8.8)
NP intensity	4.3 (2.3, 6.0)	4.4 (1.4, 7.4)	3.9 (0, 6.8)
NP frequency	9.5 (0, 14)	10 (0, 14)	7.5 (0, 14)

AUC: area under the curve (intensity × duration); PSFS: Patient-Specific Functional Scale; NP: neck pain.

**Table 4:** Effects of the intervention on headache-related outcomes, neck-pain-related outcomes, and functionality for the overall group and for each headache type.

	T1 vs. T0			T2 vs. T0			Effect Size (r)
	W	z	Sig (*)	W	z	Sig (*)	
Overall group (*n* = 22)							
Intensity	120	–0.211	0.849	122	0.226	0.835	0.048
Frequency	41.5	0.756	0.472	61	2.490	0.014 *	0.531
Duration	98	1.551	0.127	119	2.637	0.009 *	0.562
AUC	161	1.120	0.276	184	2.381	0.018 *	0.508
Medication	72	1.224	0.226	101	1.706	0.089	0.364
PSFS	20	–3.355	<0.001 *	23	–3.360	<0.001 *	−0.716
NP intensity	70	–1.307	0.202	140	1.307	0.202	0.279
NP frequency	17	–1.992	0.049 *	74	1.992	0.049 *	0.425
Migraine (*n* = 10)							
Intensity	25	–0.255	0.846	23	–0.459	0.695	−0.145
Frequency	10.5	0.809	0.490	20	1.992	0.056	0.630
Duration	15	0.169	0.933	29	1.540	0.141	0.487
AUC	19	–0.866	0.432	42	1.478	0.160	0.467
Medication	18	0.676	0.546	23.5	1.606	0.125	0.508
PSFS	5	–2.293	0.025 *	4	–2.395	0.014 *	−0.757
NP intensity	41	1.376	0.193	38	1.070	0.322	0.338
NP frequency	23	1.521	0.143	31	1.820	0.079	0.576
Tension-type headache (*n* = 10)							
Intensity	34	–0.392	0.733	40	0.622	0.563	0.180
Frequency	11.5	0.210	0.916	13	1.483	0.176	0.428
Duration	38	1.836	0.076	34	2.240	0.030 *	0.647
AUC	65	2.040	0.042 *	50	1.334	0.142	0.385
Medication	20	1.014	0.348	30	0.889	0.402	0.257
PSFS	6	–2.401	0.018 *	5	–2.667	0.008 *	−0.770
NP intensity	32	0.459	0.695	34	0.663	0.557	0.191
NP frequency	13.5	0.629	0.598	10	0.674	0.583	0.195

AUC: area under the curve (intensity × duration); PSFS: Patient-Specific Functional Scale; NP: neck pain; T0: baseline period (2 weeks before the intervention); T1: end of the supervised exercise period (weeks 7–8); T2: end of the non-supervised period (weeks 13–14). * Statistical significant within-group differences (Significance level *p*-value < 0.05).

## Data Availability

The raw data supporting the conclusions of this article will be made available by the authors upon request.

## Supplementary Materials

The following supporting information can be downloaded at https://www.mdpi.com/article/10.3390/jcm14155359/s1, Table S1: Characteristics of the 8-week program exercises; Table S2: Summary of the intervention; Figure S1: Rate of perceived exertion (RPE). Additional Content: Support guide for participants, including educational information provided and exercise diary.

## Abbreviations

The following abbreviations are used in this manuscript:

ICHD-3	*International Classification of Headache Disorders*, third edition
TTH	Tension-Type Headache
AUC	Area Under the Curve
RPE	Rate of Perceived Exertion
PSFS	Patient-Specific Functional Scale
NP	Neck Pain
BoNT/A	Onabotulinum toxin A
DHC	Danish Headache Center
