# Supplementary material for: Strength Training and Posture Correction of the Neck and Shoulder for Patients with Chronic Primary Headache: A Prospective Single-Arm Pilot Study
Source: J Clin Med. 2025 Jul 29;14(15):5359. doi: 10.3390/jcm14155359 (PMC12347830; doi:10.3390/jcm14155359)
Supplement: Supplementary file 1 [file jcm-14-05359-s001.zip › jcm-3749642-supplementary.pdf]

Supplementary material

Table S1. Characteristics of the 8-week program exercises.

<table>
<tr><th rowspan="2">Week</th><th colspan="3">1-Craniocervical flexion</th><th colspan="3">2-Cervical retraction</th><th colspan="4">3-Shoulder exercises</th></tr>
<tr><th>Level</th><th>Sets</th><th>Reps</th><th>%MR</th><th>Sets</th><th>Reps</th><th>%RM</th><th>Sets</th><th>Reps</th><th>RPE</th></tr>
<tr><td>1</td><td>1</td><td rowspan="9">3</td><td rowspan="9">10</td><td rowspan="2">50</td><td>2-3</td><td rowspan="9">15-20</td><td>50-70</td><td rowspan="9">3-4</td><td rowspan="2">8-12</td><td>6-7</td></tr>
<tr><td>2</td><td rowspan="2">2</td><td rowspan="8">3-4</td><td>70</td><td rowspan="8">Always >7/10
Never 10/10</td></tr>
<tr><td>3</td><td rowspan="2">60</td><td rowspan="2">70-80</td><td rowspan="2">8-10</td></tr>
<tr><td>4</td><td>2-3</td></tr>
<tr><td>5</td><td rowspan="5">3</td><td rowspan="5">70</td><td rowspan="5">80</td><td rowspan="5">8</td></tr>
<tr><td>6</td></tr>
<tr><td>7</td></tr>
<tr><td>8</td></tr>
<tr><td>Follow-up</td></tr>
</table>

%MR: Percentage of maximum repetition; **RPE**: Rate of perceived exertion

Table S2. Summary of the intervention.

**Postural corrections**	Description
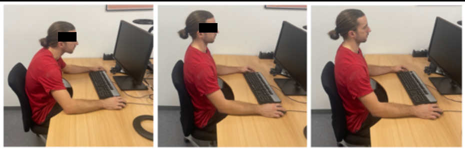	Repeat a set of 10 repetitions 3 times per day.<br>Go into bad posture: Forward head posture + slump.<br>Overcorrect the posture: Straight back, shoulders and put your head backwards.<br>Relax the posture 10%.<br>Repeat 1 set of 10 corrections three times per day.
**Exercises**	
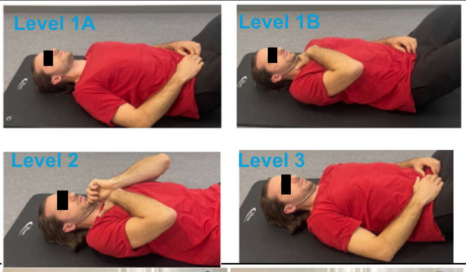	**Deep neck flexors**<br>In a lying position rotate your head over you neck. The chin approaches to the neck. The progression for this exercise is performed through 3 levels:<br>**Level 1**: Do the movement without activation of other neck muscles and hold the position 2 - 5”. Stay in this level until instructed by physio.<br>**Level 2**: Put a slight resistance using your fist and hold the position 2 - 5”.<br>**Level 3**: Separate your head from the floor and hold the position for 2 - 5”. Increase progressively (+2”).
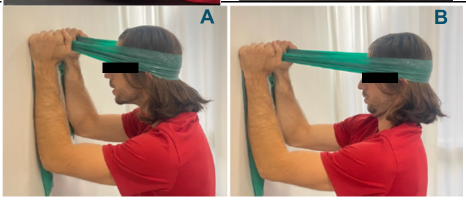	**Cervical retraction**<br>Take the band in front of you with your hands and around the head. Put your head backwards horizontally straining the elastic band. Go back forwards slowly.<br>Range: A – B – A.<br>How: Slowly and controlled.<br>3 sets of 15 to 20 reps.<br>Light to moderate intensity. RPE: 4-6/10.
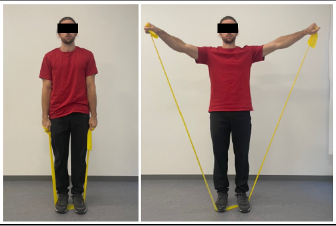	**Shoulder abduction**<br>Elevate your arms laterally.<br>It is possible to advance the arms 30º forward to avoid discomfort.<br><br>How: Pull the band fast and relax it slowly.<br>3-4 sets of 8 to 12 reps until failure.<br>RPE: >7/10
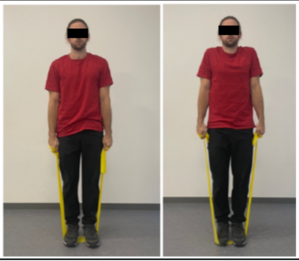	**Shrugs**<br>Elevate your shoulders without flexing your elbows. Holding the elastic band with your feet.<br><br>How: Pull the band fast and relax it slowly.<br>3-4 sets of 8 to 12 reps until failure.<br>RPE: >7/10
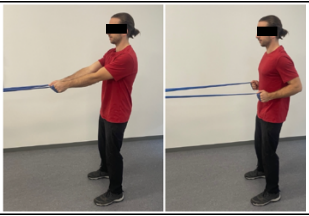	**Bilateral row**<br>Put the elastic band hold in front of you. Row with both arms at the same time to your trunk sides. It is allowed to place one feed advanced for more stability.<br>How: Pull the band fast and relax it slowly.<br>3-4 sets of 8 to 12 reps until failure.<br>RPE: >7/10
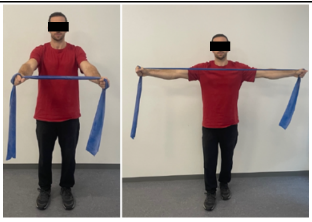	**Horizontal abduction**<br>Hold the elastic band on one side and pull backwards holding from the opposite arm. Your body should be positioned horizontal to the band anchor.<br><br>How: Pull the band fast and relax it slowly.<br>3-4 sets of 8 to 12 reps until failure.<br>RPE: >7/10

Score	Effort
10	Maximal
9	Very strong
7-8	Strong
5-6	Moderate
3-4	Light
1-2	Very light
0	Absolute resting

Figure S1. Rate of perceived exertion (RPE). Patients were instructed to achieve at least 7/10 since 2nd week of the training and avoid 10/10 “Maximal efforts”.

Additional Content: Support guide to participants

STRENGTH TRAINING AND POSTURE CORRECTION FOR HEADACHE

NAVN:_________________________________

Instruktør: Jordi Padrós Augé, physiotherapist, PhD studerende.

Tilsynsførende: Bjarne Kjeldgaard Madsen

Index

Content of the guide	**Page 3**
Exercise intensity, muscle function and headaches	**Page 4**
Tips and tricks *(to improve muscle strength)*	**Page 5**
Ergonomics & Posture	**Page 6**
List of exercises	**Page 7-11**
Summary of exercises	**Page 12**
Exercise plan	**Pages 13-21**
week 1	Page 13
week 2	Page 14
week 3	Page 15
week 4	Page 16
week 5	Page 17
week 6	Page 18
week 7	Page 19
week 8	Page 20
Summary of exercise plan	Page 21
Additional information	**Page 22**
Contact information	**Page 23**

Content of the guide

This guide aims to provide you with the necessary information to understand why you train and help you carry out the program prescribed by the physiotherapist at home.

You will find information on how to follow the exercise plan on Pages 4-5. This includes information about the purpose of training, posture correction, and headaches.

On pages 7–11, you will find detailed information on the exercises of the program, such as pictures and descriptions.

On pages 13–20, you will find your exercise plan. On each week plan, you will find two tables, one with the description of the exercises, and the other for registration after each session. A feedback scale to determine the intensity level of the session was also provided. This section is very important to complete after the sessions, as it will help us adjust the exercises weekly.

Finally, on page 22, you find a few instructions to continue training after the course because the benefits of exercise are perceived as much as exercise is performed. This course also aimed to teach your skills to become good at exercising.

In summary, we believe that this guide provides all the information necessary to perform **daily postural corrections** and **three strength training sessions per week**.

<u>Exercise resistance, muscle function and headaches</u>

Why resistance matters?

To become stronger the exercises should be performed with enough resistance.

What is the relation between my neck and shoulder muscles and my headache?

Neck and shoulder muscle capacity allows us to cope with the demands of daily life, e.g., maintain positions, deal with different tasks, etc. When demands are higher than muscles can handle it causes pain.

Postural corrections (decrease demands on muscles) combined with strength training of the neck and shoulder muscles (increase capacity of the muscles). Further we aim to improve muscle function of the neck and shoulder muscles.

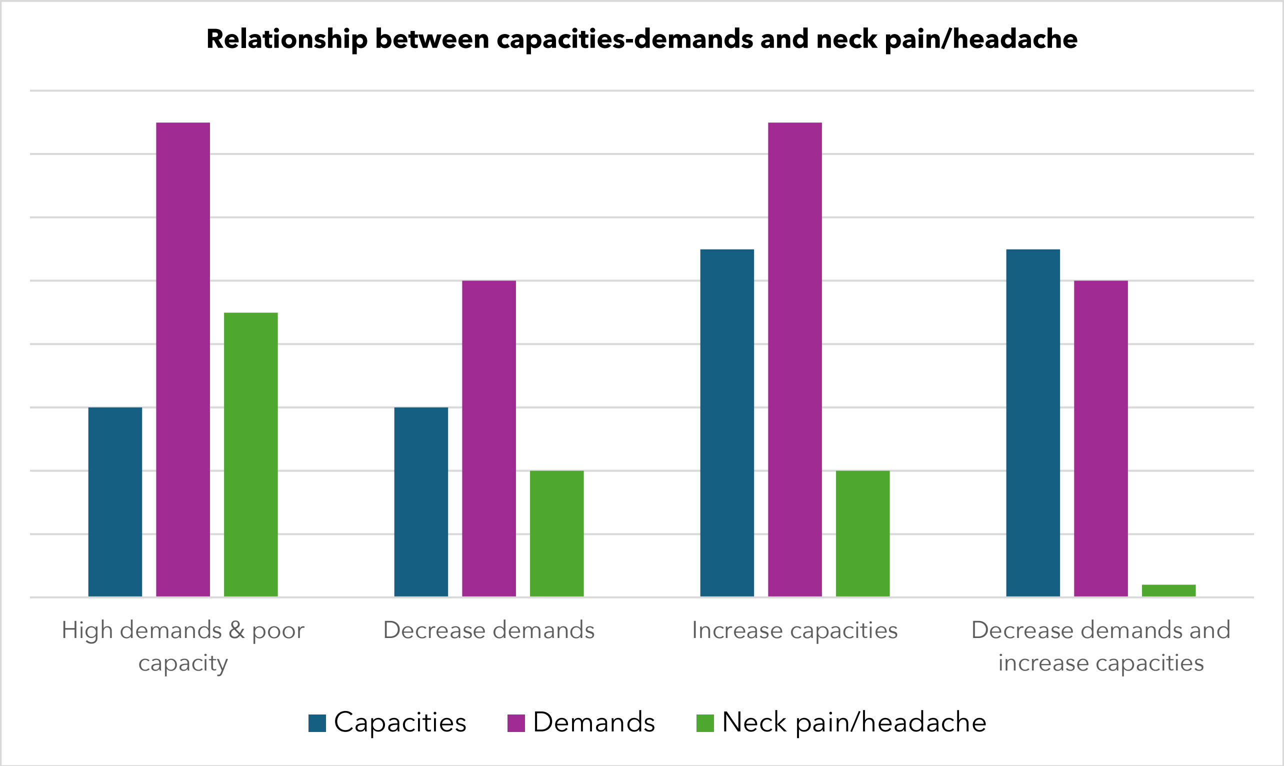

The neck and shoulders regions have a strong connection with the sensitive nerves of the head and face, that's why the relation between muscle capacities and demands of these areas potentially can influence neck pain and headache.

We aim to give you stronger muscles through training; and less load on muscles through better posture, and as consequence, decrease pain.

<u>Tips and tricks</u> *(to improve muscle strength)*

How to get stronger muscles?

Improving muscle capacity depends on how we exercise the muscles. Training at high resistance helps obtain stronger muscles faster than other types of training. Therefore, we aim to achieve **muscle failure** during each session.

What muscle failure means?

Muscle failure means that we are unable to perform more repetitions of an exercise at one point. To achieve failure safely, the <u>ideal condition is that failure occurs between 8 and 12 repetitions and that it is not possible to perform more than 3 sets with that resistance.</u>

First of all, failure shouldn't happen before the repetition number 8 or after repetition number 12 of the first set, that will mean that the resistance is too strong or too weak respectively.

- If failure does not occur before rep 12, then this means that the resistance is too weak, so is a good practice to do add resistance.
- If the failure occurs before the repetition 8 in the first set, is a good practice to decrease the resistance a little.
- In the 3rd set, if the failure occurs before repetition 8 is also good, that means you perform a nice training.

Tricks to increase resistance *(the order presented is important)***:**

- <u>Less rest between exercises</u>. You can try to perform each exercise with 30 seconds rest instead of a minute.
- <u>Adding more resistance</u>. Use a combination of two or more bands if needed. Below the resistance of each color is provided.

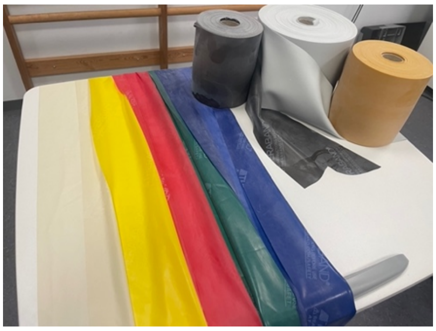

<u>From low to high resistance:</u>

White – Yellow – Red – Green – Blue – Black – Silver – Gold

<u>Ergonomics and posture corrections</u>

Some type of postures and sustain any posture for long time period is high demanding for our muscles and can cause pain, and specifically for the neck and shoulder muscles. It is important to reduce the load on these muscles.

First tip: Ergonomic adjustments should be considered in work-sustained tasks because continuous muscle activity can cause muscle pain. Each job has its own adjustments (e.g., laboratory testing, desk office work) that can easily be solved by changing position. This support can be discussed in the course with the physiotherapist.

Second tip: Maintaining "bad posture" increase the relative load on the muscles. Breaking sustained postures is useful for oxygenating muscles and reducing overactivity.

Third tip: Within the positions held, there were some better than others. Small postural adaptations can result in significant changes in muscle demands. Correcting the forward head position, putting your back straight and shoulders back, and then relaxing that position by 10% (Do 10 reps at least 3 times/day) is very useful throughout the workday (pictures).

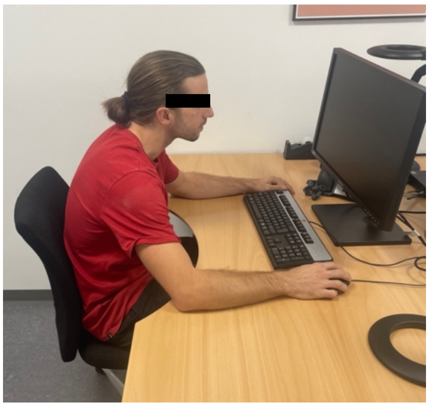

Go into bad posture

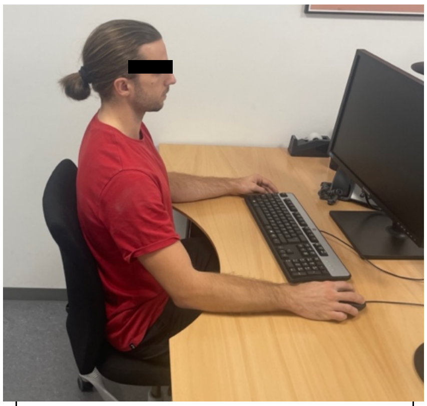

Overcorrection

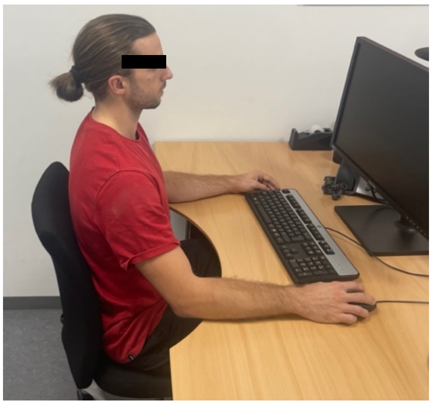

Relax 10%

Recommendation: Do 10 reps at least 3 times per day.

List of exercises

NECK EXERCISES

1- Deep Neck Flexors

In a lying position rotate your head over you neck. The chin approaches to the neck. Do not activate the superficial neck muscles, put 3 fingers on your neck to feel the activity control.

Level 1. Do the movement "slightly yes" without activation of other neck muscles and hold the position for 2 to 5".

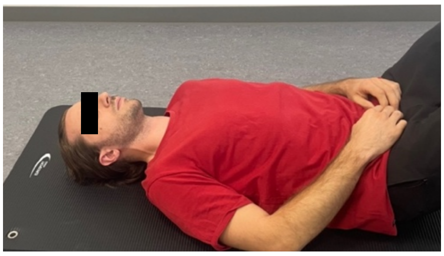
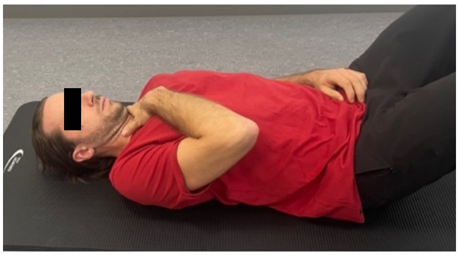

Level 2. Put a slight resistance using your fist or thumb on your chin and hold the position 2-5" without activity of the superficial neck muscles.

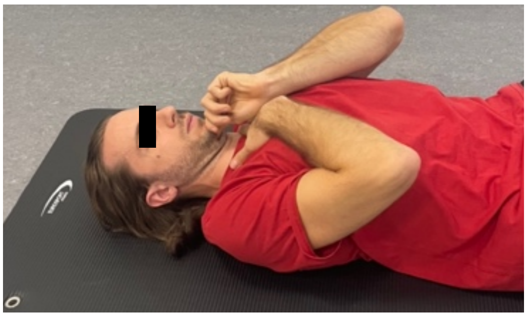

Level 3. From the level 1, without separating the chin from the neck, separate the head from the surface and hold the position for 2 to 5".

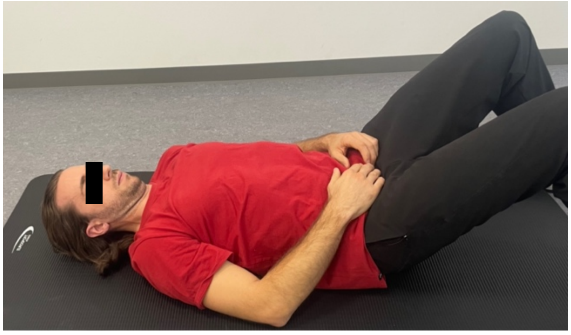

2- Neck retraction

Put your head backwards horizontally against the elastic band. Took the band in front of you with your hands. Go back forwards slowly.

Option A. Holding the band with your hands on a wall.

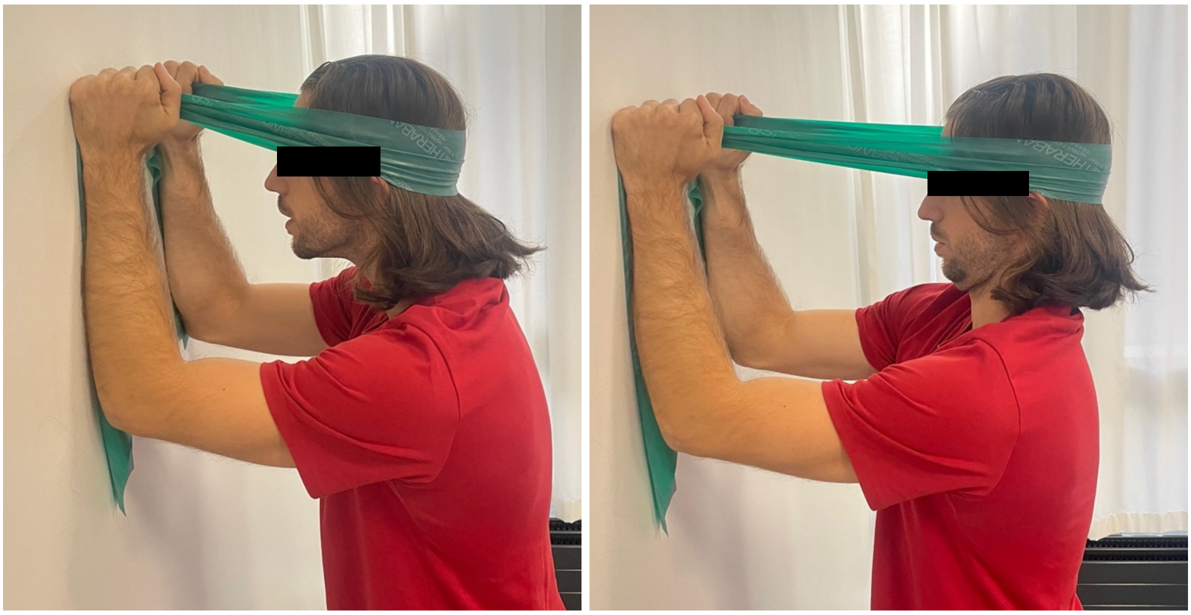

Option B. With the elastic band strapped.

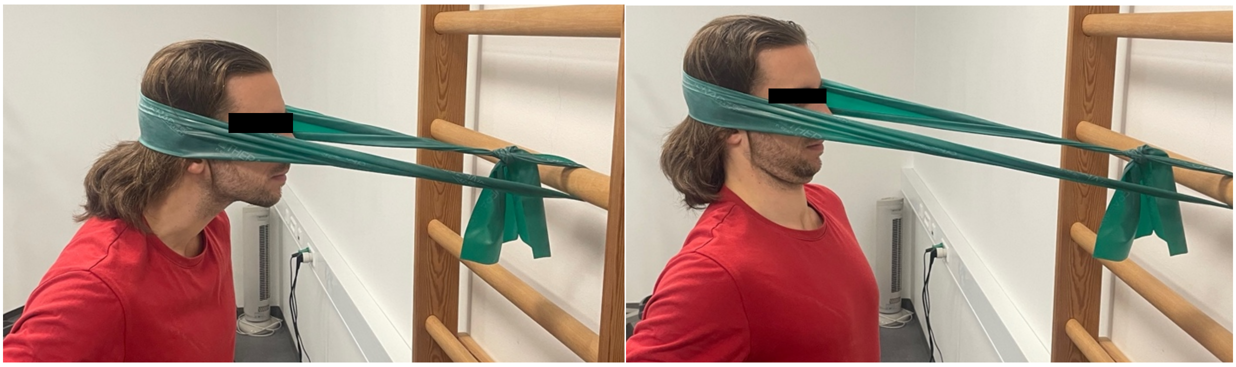

SHOULDER EXERCISES

3- Shoulder abduction

Elevate your arms laterally with the elastic band hold by your feet.
Option A. Standing

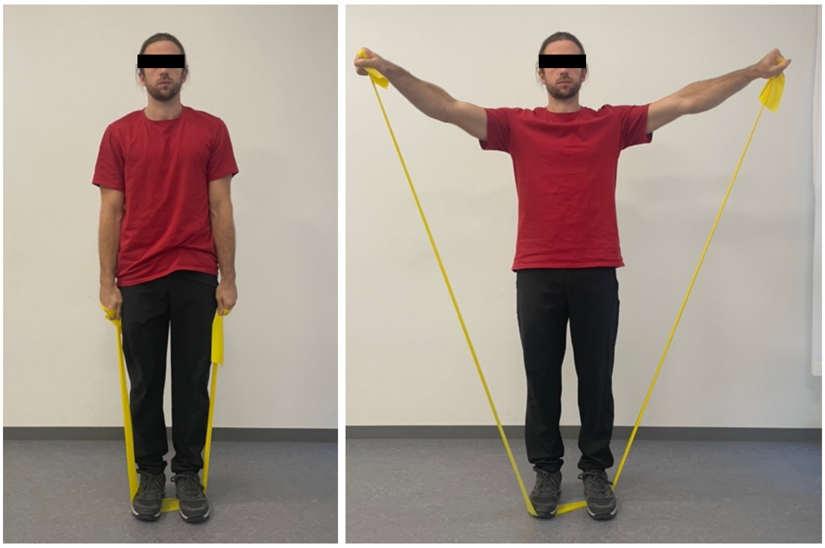

Option B. Lying

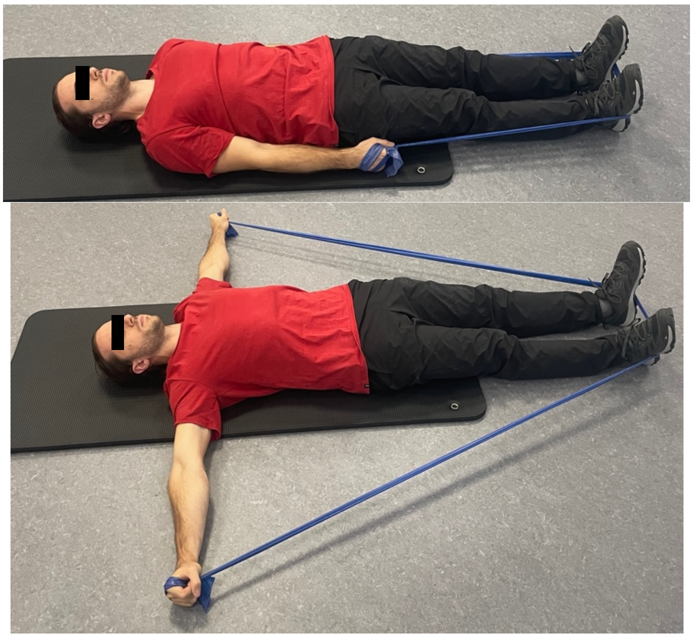

4- Shoulder elevation

Elevate your shoulders without flexing your elbows. Hold the elastic band with your feet.
Option A. Standing

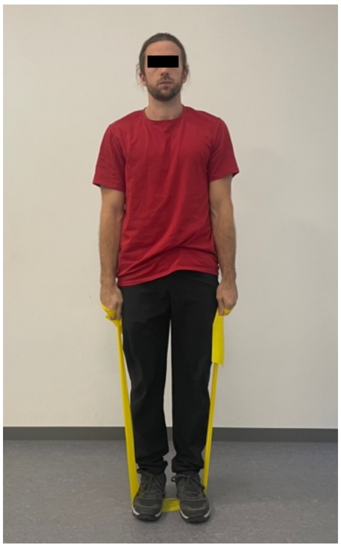
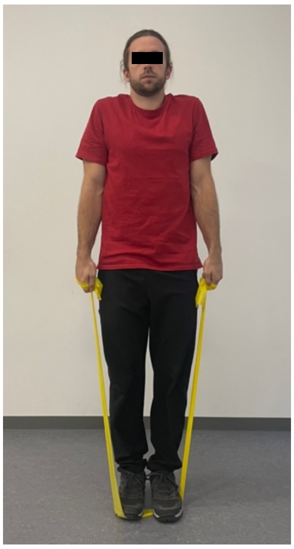

Option B. Lying

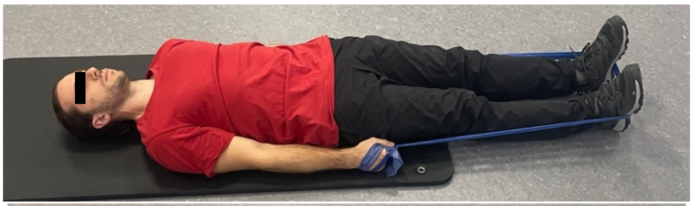
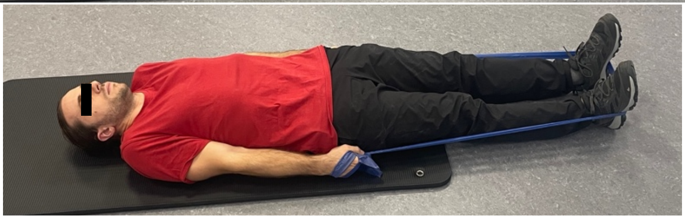

5- Horizontal row

Put the elastic band hold in front of you. Row with both arms at the same time to your trunk sides.

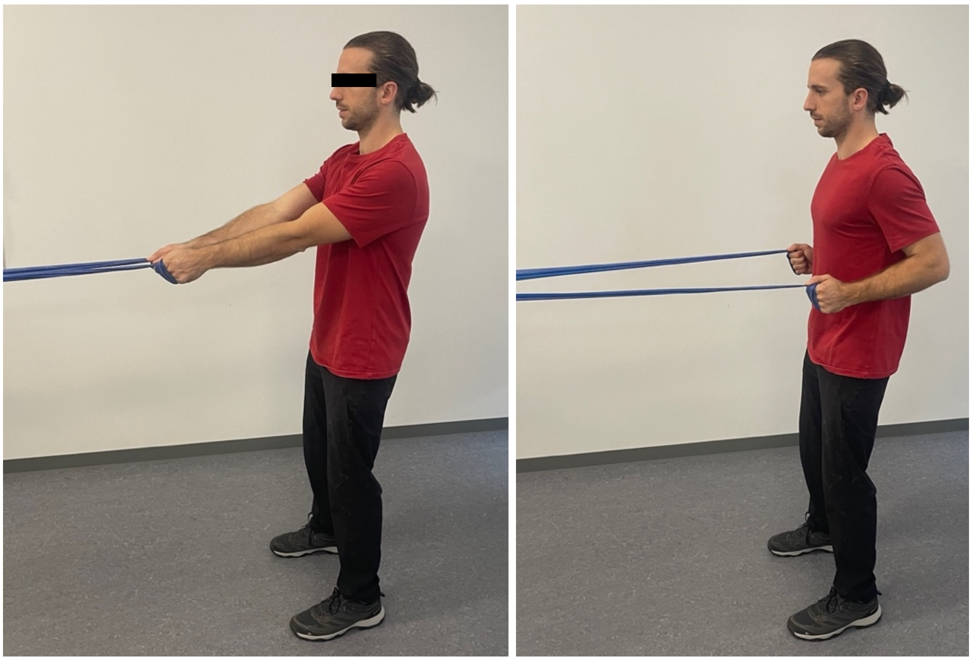

6- Horizontal abduction

Hold the elastic band on one side and pull backwards holding from the opposite arm. Your body should be positioned horizontal to the band anchor.

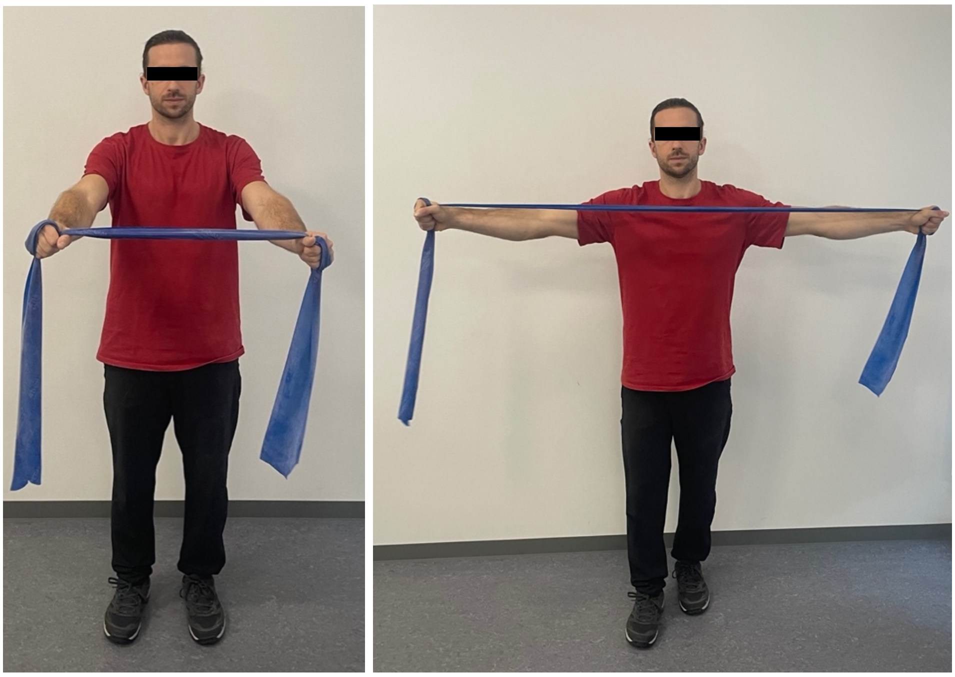

Summary of exercises

Name and pictures	Description of the exercise
**1. Deep neck flexion**<br>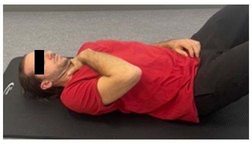	In a lying position rotate your head over you neck. The chin approaches to the neck. The progression for this exercise is performed through 3 levels:<br>**Level 1:** Do the movement without activation of other neck muscles and hold the position 2 - 5". Stay in this level until instructed by physio.<br>**Level 2:** Put a slight resistance using your fist and hold the position 2 - 5".<br>**Level 3:** Separate your head from the floor and hold the position for 2 - 5".
**2. Neck retraction**<br>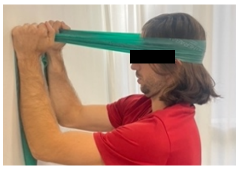	Put your head backwards horizontally straining the elastic band. Took the band in front of you with your hands. Go back forwards slowly.<br><br>How: Slowly and controlled
**3. Shoulder abduction**<br>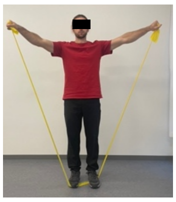	Elevate your arms in front of you around 30° separated with the elastic band hold by your feet.<br><br>How: Until failure.
**4. Shoulder elevation**<br>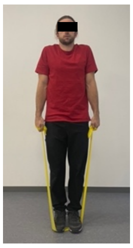	Elevate your shoulders without flexing your elbows. Hold the elastic band with your feet.<br><br>How: Until failure.
**5. Horizontal row**<br>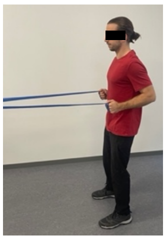	Put the elastic band hold in front of you. Row with both arms at the same time to your trunk sides.<br><br>How: Until failure.
**6. Horizontal abduction**<br>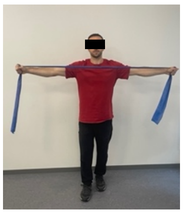	Hold the elastic band on one side and pull backwards holding from the opposite arm. Your body should be positioned horizontal to the band anchor.<br><br>How: Until failure.

Exercise plan - **Week 1**

**Exercise 1**	**Pictures**	**Level**	**Sets**	**Reps**	**Rest between sets**
1. Deep neck flexors	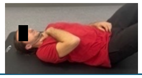	1	3	10	30"-60"
**Exercise 2 to 6**		**Band color**	**Sets**	**Reps**	**Rest between sets**
2. Neck retraction	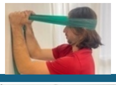		3-4	15-20	1-2'
3. Shoulder abduction	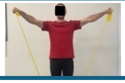		3-4	The **failure** should occur on **rep 12***.	1-2'
4. Shoulder elevation	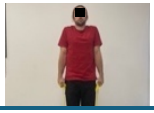				
5. Horizontal row	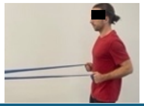				
6. Horizontal abduction	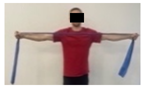				

**Score**	**Effort**
**10**	Maximal
**9**	Very strong
**7-8**	Strong
**5-6**	Moderate
**3-4**	Light
**1-2**	Very light
**0**	Absolute resting

Postural corrections

Date	Times performed		
	☐	☐	☐
	☐	☐	☐
	☐	☐	☐
	☐	☐	☐
	☐	☐	☐
	☐	☐	☐
	☐	☐	☐

Reporting (please, full fill out on each session)

	Pain before exercise session (0-10)		Place tick "X"		Pain after exercise session (0-10)		Date	**Reporting effort** (See table above)
	Headache/Migraine	Neck pain	Home	DHC	Headache/Migraine	Neck pain		
Session 1								
Session 2								
Session 3								

*The number of repetitions is the estimated number of repetitions to achieve the **failure**.

Exercise plan - **Week 2**

**Exercise 1**	**Pictures**	**Level**	**Sets**	**Reps**	**Rest between sets**
1. Deep neck flexors	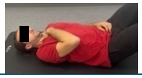		3	10	30-60″
**Exercise 2 to 6**		**Band color**	**Sets**	**Reps**	**Rest between sets**
2. Neck retraction	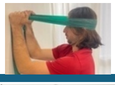		3-4	15-20	1-2′
3. Shoulder abduction	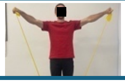		3-4	The **failure** should occur on **reps 10-12***.	1-2′
4. Shoulder elevation	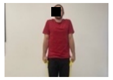				
5. Horizontal row	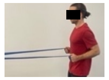				
6. Horizontal abduction	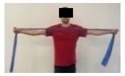				

**Score**	**Effort**
**10**	Maximal
**9**	Very strong
**7-8**	Strong
**5-6**	Moderate
**3-4**	Light
**1-2**	Very light
**0**	Absolute resting

Postural corrections

Date	Times performed		
	☐	☐	☐
	☐	☐	☐
	☐	☐	☐
	☐	☐	☐
	☐	☐	☐
	☐	☐	☐
	☐	☐	☐

Reporting (please, full fill out on each session)

	Pain before exercise session (0-10)		Place tick "X"		Pain after exercise session (0-10)		Date	**Reporting effort** (See table above)
	Headache/Migraine	Neck pain	Home	DHC	Headache/Migraine	Neck pain		
Session 4								
Session 5								
Session 6								

*The number of repetitions is the estimated number of repetitions to achieve the **failure**.

Exercise plan - **Week 3**

Exercise 1	Pictures	Level	Sets	Rest between sets
1. Deep neck flexors	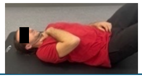		3	30-60″

Exercise 2 to 6		Band color	Sets	Reps	Rest between sets
2. Neck retraction	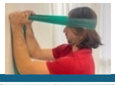		3-4		1-2′
3. Shoulder abduction	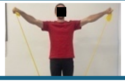		3-4	The **failure** should occur on **reps 8-10***.	1-2′
4. Shoulder elevation	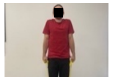				
5. Horizontal row	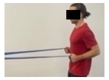				
6. Horizontal abduction	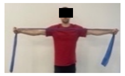				

Score	Effort
**10**	Maximal
**9**	Very strong
**7-8**	Strong
**5-6**	Moderate
**3-4**	Light
**1-2**	Very light
**0**	Absolute resting

Postural corrections

Date	Times performed		
	☐	☐	☐
	☐	☐	☐
	☐	☐	☐
	☐	☐	☐
	☐	☐	☐
	☐	☐	☐
	☐	☐	☐

Reporting (please, full fill out on each session)

	Pain before exercise session (0-10)		Place tick "X"		Pain after exercise session (0-10)		Date	**Reporting effort** (See table above)
	Headache/Migraine	Neck pain	Home	DHC	Headache/Migraine	Neck pain		
Session 7								
Session 8								
Session 9								

*The number of repetitions is the estimated number of repetitions to achieve the **failure**.

Exercise plan - **<u>Week 4</u>**

**Exercise 1**	**Pictures**	**Level**	**Sets**	**Reps**	**Rest between sets**
1. Deep neck flexors	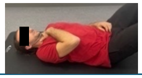	1	3	10	30-60″
**Exercise 2 to 6**		**Band color**	**Sets**	**Reps**	**Rest between sets**
2. Neck retraction	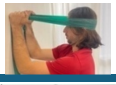		3-4	15-20	1-2′
3. Shoulder abduction	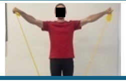		3-4	The **failure** should occur on **reps 8***.	1-2′
4. Shoulder elevation	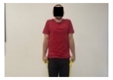				
5. Horizontal row	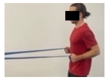				
6. Horizontal abduction	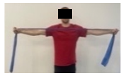				

**Score**	**Effort**
**10**	Maximal
**9**	Very strong
**7-8**	Strong
**5-6**	Moderate
**3-4**	Light
**1-2**	Very light
**0**	Absolute resting

Postural corrections

Date	Times performed
	☐ ☐ ☐
	☐ ☐ ☐
	☐ ☐ ☐
	☐ ☐ ☐
	☐ ☐ ☐
	☐ ☐ ☐
	☐ ☐ ☐

Reporting (please, full fill out on each session)

	Pain before exercise session (0-10)		Place tick "X"		Pain after exercise session (0-10)		Date	**Reporting effort** (See table above)
	Headache/Migraine	Neck pain	Home	DHC	Headache/Migraine	Neck pain		
Session 10								
Session 11								
Session 12								

*The number of repetitions is the estimated number of repetitions to achieve the **failure**.

Exercise plan - **Week 5**

Exercise 1	Pictures	Level	Sets	Reps	Rest between sets
1. Deep neck flexors	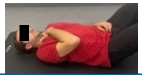	1	3	10	30”-60”
**Exercise 2 to 6**		**Band color**	**Sets**	**Reps**	**Rest between sets**
2. Neck retraction	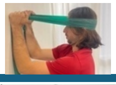		3-4	15-20	1-2’
3. Shoulder abduction	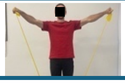		3-4	The **failure** should occur on **reps 8***.	1-2’
4. Shoulder elevation	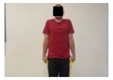				
5. Horizontal row	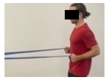				
6. Horizontal abduction	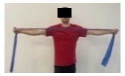				

Score	Effort
**10**	Maximal
**9**	Very strong
**7-8**	Strong
**5-6**	Moderate
**3-4**	Light
**1-2**	Very light
**0**	Absolute resting

Postural corrections

Date	Times performed		
	☐	☐	☐
	☐	☐	☐
	☐	☐	☐
	☐	☐	☐
	☐	☐	☐
	☐	☐	☐
	☐	☐	☐

Reporting (please, full fill out on each session)

	Pain before exercise session (0-10)		Place tick “X”		Pain after exercise session (0-10)		Date	**Reporting effort** (See table above)
	Headache/Migraine	Neck pain	Home	DHC	Headache/Migraine	Neck pain		
Session 13								
Session 14								
Session 15								

*The number of repetitions is the estimated number of repetitions to achieve the **failure**.

Exercise plan - **Week 6**

Exercise 1	Pictures	Level	Sets	Reps	Rest between sets
1. Deep neck flexors	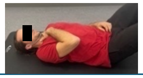		3	10	30-60″
**Exercise 2 to 6**		**Band color**	**Sets**	**Reps**	**Rest between sets**
2. Neck retraction	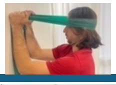		3-4	15-20	1-2′
3. Shoulder abduction	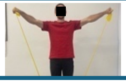		3-4	The **failure** should occur on **reps 8***.	1-2′
4. Shoulder elevation	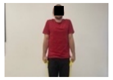				
5. Horizontal row	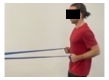				
6. Horizontal abduction	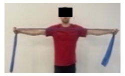				

Score	Effort
**10**	Maximal
**9**	Very strong
**7-8**	Strong
**5-6**	Moderate
**3-4**	Light
**1-2**	Very light
**0**	Absolute resting

Postural corrections

Date	Times performed		
	☐	☐	☐
	☐	☐	☐
	☐	☐	☐
	☐	☐	☐
	☐	☐	☐
	☐	☐	☐
	☐	☐	☐

Reporting (please, full fill out on each session)

	Pain before exercise session (0-10)		Place tick "X"		Pain after exercise session (0-10)		Date	**Reporting effort** (See table above)
	Headache/Migraine	Neck pain	Home	DHC	Headache/Migraine	Neck pain		
Session 16								
Session 17								
Session 18								

*The number of repetitions is the estimated number of repetitions to achieve the **failure**.

Exercise plan - **<u>Week 7</u>**

**Exercise 1**	**Pictures**	**Level**	**Sets**	**Reps**	**Rest between sets**
1. Deep neck flexors	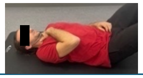		3	10	30-60"
**Exercise 2 to 6**		**Band color**	**Sets**	**Reps**	**Rest between sets**
2. Neck retraction	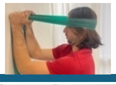		3-4	15-20	1-2'
3. Shoulder abduction	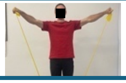		3-4	The **failure** should occur on **reps 8***.	1-2'
4. Shoulder elevation	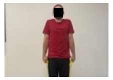				
5. Horizontal row	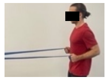				
6. Horizontal abduction	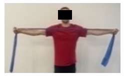				

**Score**	**Effort**
**10**	Maximal
**9**	Very strong
**7-8**	Strong
**5-6**	Moderate
**3-4**	Light
**1-2**	Very light
**0**	Absolute resting

Postural corrections

Date	Times performed		
	☐	☐	☐
	☐	☐	☐
	☐	☐	☐
	☐	☐	☐
	☐	☐	☐
	☐	☐	☐
	☐	☐	☐

Reporting (please, full fill out on each session)

	Pain before exercise session (0-10)		Place tick "X"		Pain after exercise session (0-10)		Date	**Reporting effort** (See table above)
	Headache/Migraine	Neck pain	Home	DHC	Headache/Migraine	Neck pain		
Session 19								
Session 20								
Session 21								

*The number of repetitions is the estimated number of repetitions to achieve the **failure**.

Exercise plan - **Week 8**

Exercise 1	Pictures	Level	Sets	Reps	Rest between sets
1. Deep neck flexors	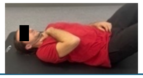		3	10	30-60″
**Exercise 2 to 6**		**Band color**	**Sets**	**Reps**	**Rest between sets**
2. Neck retraction	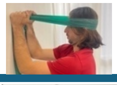		3-4	15-20	1-2′
3. Shoulder abduction	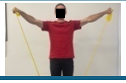		3-4	The **failure** should occur on **reps 8***.	1-2′
4. Shoulder elevation	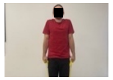				
5. Horizontal row	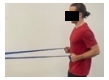				
6. Horizontal abduction	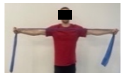				

Score	Effort
**10**	Maximal
**9**	Very strong
**7-8**	Strong
**5-6**	Moderate
**3-4**	Light
**1-2**	Very light
**0**	Absolute resting

Postural corrections

Date	Times performed		
	☐	☐	☐
	☐	☐	☐
	☐	☐	☐
	☐	☐	☐
	☐	☐	☐
	☐	☐	☐
	☐	☐	☐

Reporting (please, full fill out on each session)

	Pain before exercise session (0-10)		Place tick "X"		Pain after exercise session (0-10)		Date	**Reporting effort** (See table above)
	Headache/Migraine	Neck pain	Home	DHC	Headache/Migraine	Neck pain		
Session 22								
Session 23								
Session 24								

*The number of repetitions is the estimated number of repetitions to achieve the **failure**.

Summary

	Deep neck flexors (1)			Retraction (2)		Shoulder exercises (3-6)	
Week	Level	Sets	Reps	Sets	Reps	Sets	Reps
1	1	3	10	2-3	15-20	3-4	12
2	1-2	3		3-4	15-20		10-12
3	2	2-3					8-10
4	2-3	2-3					8
5	3	3					
6							
7							
8							

Band color/Level for each exercise x week

List of exercises	Week 1	Week 2	Week 3	Week 4	Week 5	Week 6	Week 7	Week 8
1. Deep neck flexors	1							
2. Retraction								
3. Abduction								
4. Elevation								
5. H. Abduction								
6. H. Row								

After course

What to do after this course?

This course aimed to study the effectiveness of a physical intervention combining exercises for headaches. Unless your headache has worsened, we encourage you to continue working on performing the exercises in the way you have learned.

Exercise has a continuous effect on the body, and as long as we continue to do it.

If you still have not seen a significant change in 8 weeks, do not worry, as long as your headache has not worsened, it is suggested that it continues to improve, and the effect may emerge in the next few weeks. Some people take longer to perceive the effects of exercise; others perceive these effects much sooner; each person has their own time.

After 20 weeks of adequate performance, you will have sufficient information to balance the benefits of exercise. Maybe not all of the exercises will provide benefits, but probably at that point you identified which ones are helping or could help you, and which ones no. Thus, the knowledge and the acquired experience will enable you to make the right decision about continuing or not with exercise and how to continue.

Please, report for each week how many days you performed strength exercises and postural correction

Week		Exercise sessions			Postural correction			
2023	51	1 ☐	2 ☐	3 ☐	Never ☐	1-2 days ☐	3-4 days ☐	every day ☐
	52	1 ☐	2 ☐	3 ☐	Never ☐	1-2 days ☐	3-4 days ☐	every day ☐
2024	1	1 ☐	2 ☐	3 ☐	Never ☐	1-2 days ☐	3-4 days ☐	every day ☐
	2	1 ☐	2 ☐	3 ☐	Never ☐	1-2 days ☐	3-4 days ☐	every day ☐
	3	1 ☐	2 ☐	3 ☐	Never ☐	1-2 days ☐	3-4 days ☐	every day ☐
	4	1 ☐	2 ☐	3 ☐	Never ☐	1-2 days ☐	3-4 days ☐	every day ☐

Contact information

Can you contact the research team?

The research team is at your disposal to answer questions regarding this project, and we will be happy to resolve them through the contact information provided on the next page.

Contact mails:
Physiotherapist: jordi.padros.auge@regionh.dk; jpadros@umanresa.cat
Supervisor: Bjarne.kjeldgaard.madsen@regionh.dk

Phone number:

+45 38 63 20 62 (Danish Headache Center)

+34 613 040 826 (Jordi. It's ok to use WhatsApp)

Address:

Dansk Hovedpinecenter Valdemar Hansens Vej, 2600 Glostrup– Danmark.
